# Supplementary figures and images for: A novel nomogram to predict the risk of requiring mechanical ventilation in patients with sepsis within 48 hours of admission: a retrospective analysis
Source: PeerJ. 2024 Nov 1;12:e18500. doi: 10.7717/peerj.18500 (PMC11533908; doi:10.7717/peerj.18500)

**A**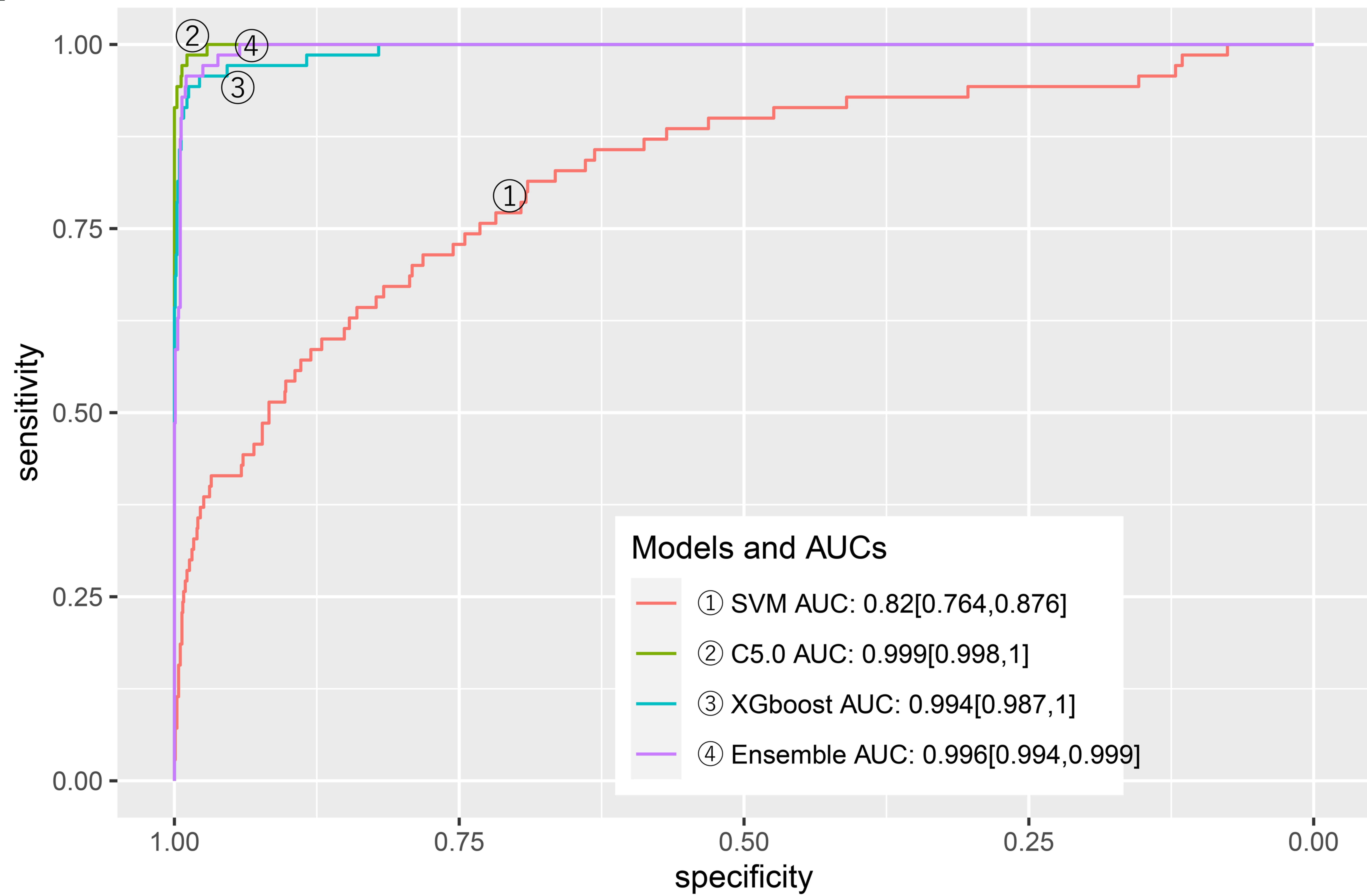**B**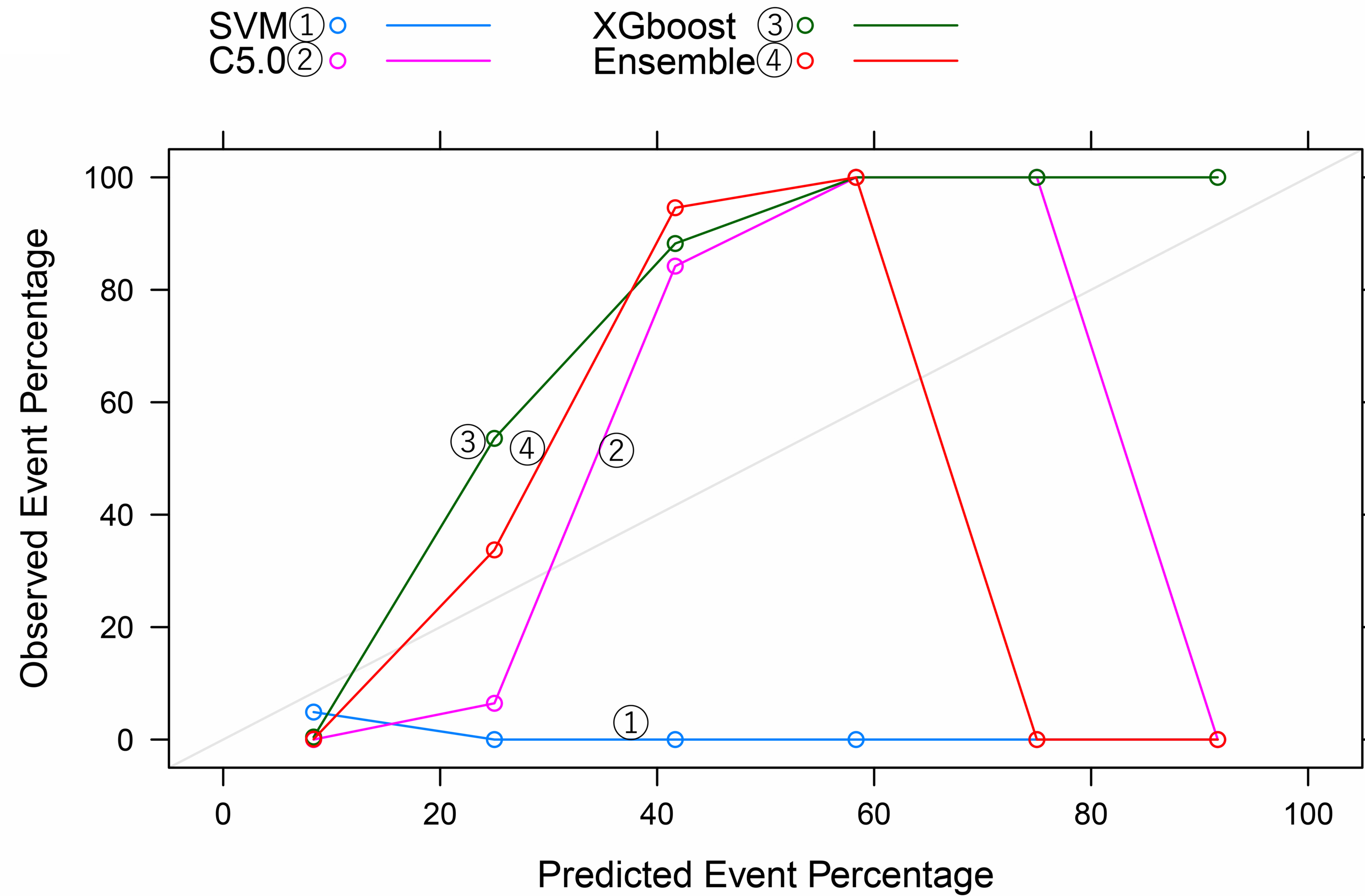

Supplement: Supplemental Information 1 [file peerj-12-18500-s001.pdf]

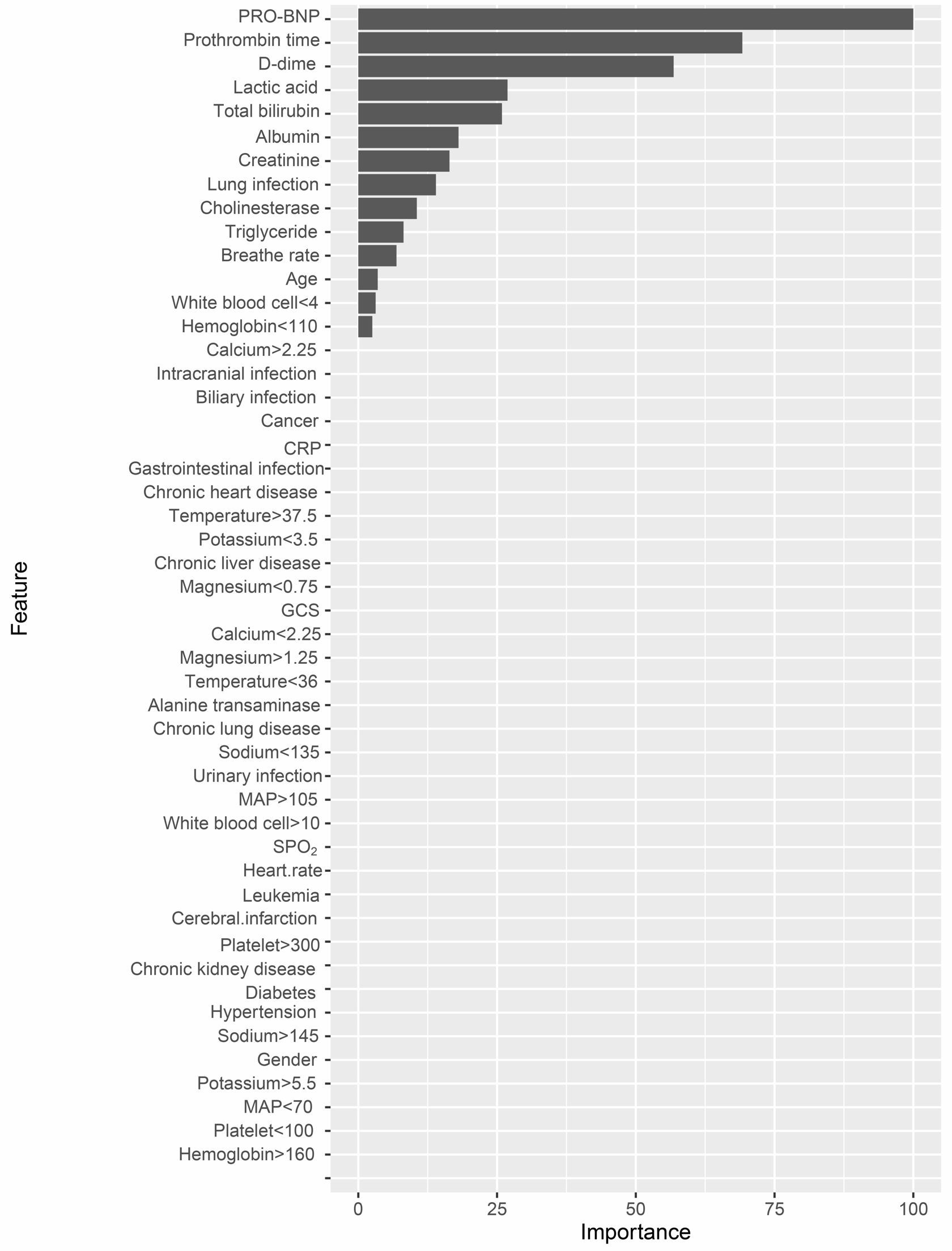

Supplement: Supplemental Information 2 [file peerj-12-18500-s002.pdf]
