# Supplementary material for: A novel nomogram to predict the risk of requiring mechanical ventilation in patients with sepsis within 48 hours of admission: a retrospective analysis
Source: PeerJ. 2024 Nov 1;12:e18500. doi: 10.7717/peerj.18500 (PMC11533908; doi:10.7717/peerj.18500)
Supplement: Supplemental Information 3 [file peerj-12-18500-s003.docx]

| Variables | VIFs |
| --- | --- |
| Cholinesterase (U/L) | 1.992 |
| Albumin (g/L) | 1.841 |
| Triglyceride (mmol/L) | 1.339 |
| Pro-bnp (pg/ml) | 1.338 |
| Creatinine (umol/L) | 1.296 |
| D-dimer (mg/L) | 1.144 |
| Lactic acid (mmol/L) | 1.132 |
| Platelet<100 (*10^-9^/L) | 1.131 |
| Prothrombin time (s) | 1.121 |
| Platelet>300 (*10^-9^/L) | 1.040 |
| Breathe rate (times/min) | 1.036 |
| Lung infection | 1.030 |

**Supplementary table 1. The VIFs of variables in** **modeling group.**

Callout: Pro-bnp, pro-brain natriuretic peptide.
