# Supplementary material for: A novel nomogram to predict the risk of requiring mechanical ventilation in patients with sepsis within 48 hours of admission: a retrospective analysis
Source: PeerJ. 2024 Nov 1;12:e18500. doi: 10.7717/peerj.18500 (PMC11533908; doi:10.7717/peerj.18500)
Supplement: Supplemental Information 4 [file peerj-12-18500-s004.docx]

**Supplementary table 2: The P values between variables and logitp by boxTidwell test**.

| Variables | P |
| --- | --- |
| Triglyceride (mmol/L) | 0.631 |
| Creatinine (umol/L) | 0.100 |
| Lactic acid (mmol/L) | 0.340 |
| Pro-bnp (pg/ml) | 0.561 |
| Cholinesterase (U/L) | 0.268 |
| Prothrombin time(s) | 0.161 |
| D-dimer (mg/L) | 0.068 |
| Albumin (g/L) | 0.554 |
| Breathe rate (times/min) | 0.570 |

Callout: Pro-bnp, pro-brain natriuretic peptide.
